# Etablierung der Genomischen Erreger-Surveillance zur Stärkung des Pandemie- und Infektionsschutzes in Deutschland

**DOI:** 10.1007/s00103-023-03680-w

**Published:** 2023-02-22

**Authors:** Simone Scheithauer, Alexander Dilthey, Anna Bludau, Sandra Ciesek, Victor Corman, Tjibbe Donker, Tim Eckmanns, Richard Egelkamp, Hajo Grundmann, Georg Häcker, Martin Kaase, Berit Lange, Alexander Mellmann, Martin Mielke, Mathias Pletz, Bernd Salzberger, Andrea Thürmer, Andreas Widmer, Lothar H. Wieler, Thorsten Wolff, Sören Gatermann, Torsten Semmler

**Affiliations:** 1 Institut für Krankenhaushygiene und Infektiologie, Universitätsmedizin Göttingen (UMG), Georg-August Universität Göttingen, Robert-Koch-Str. 40, 37075 Göttingen, Deutschland; 2 Medizinische Mikrobiologie und Krankenhaushygiene, Universitätsklinikum Düsseldorf, Düsseldorf, Deutschland; 3 Institut für Medizinische Virologie, Universitätsklinikum Frankfurt, Frankfurt am Main, Deutschland; 4 Institut für Virologie, Charité Universitätsmedizin Berlin, Berlin, Deutschland; 5 Institut für Infektionsprävention und Krankenhaushygiene, Universitätsklinikum Freiburg, Freiburg, Deutschland; 6 Robert Koch-Institut, Berlin, Deutschland; 7 Next Generation Sequencing, Niedersächsisches Landesgesundheitsamt, Hannover, Deutschland; 8 Institut für Medizinische Mikrobiologie und Hygiene, Universitätsklinikum Freiburg, Freiburg, Deutschland; 9 Abteilung Epidemiologie, Helmholtz-Zentrum für Infektionsforschung, Braunschweig, Deutschland; 10 Institut für Hygiene, Universitätsklinikum Münster, Münster, Deutschland; 11 Institut für Infektionsmedizin und Krankenhaushygiene, Universitätsklinikum Jena, Jena, Deutschland; 12 Infektiologie, Abteilung für Krankenhaushygiene und Infektiologie, Universitätsklinikum Regensburg, Regensburg, Deutschland; 13 Abteilung für Infektiologie und Spitalhygiene, Universitätsspital Basel, Basel, Schweiz; 14 Institut für Hygiene und Mikrobiologie, Ruhr-Universität Bochum, Bochum, Deutschland

**Keywords:** Strategie, Netzwerk, Best-Practice, Rahmenwerk, Standardisierung, Strategy, Network, Best practice, Framework, Standardisation

## Abstract

Die SARS-CoV-2-Pandemie hat ein Defizit an essentieller infektionsepidemiologischer Infrastruktur, insbesondere in Bezug auf die Genomische Erreger-Surveillance (GES) in Deutschland, gezeigt. Zur Vorbereitung auf zukünftige pandemische Notlagen sehen es die Autor*innen als dringend erforderlich an, dieses bestehende Defizit durch den Aufbau einer leistungsfähigen Infrastruktur für GES zu beheben. Ein derartiges Netzwerk kann auf bereits regional initiierten Strukturen, Prozessen und Interaktionen aufbauen und diese weiter optimieren. Es kann zukünftig mit einer hohen Anpassungsfähigkeit auf aktuelle und kommende Herausforderungen reagieren.

Ziele der vorliegenden Arbeit sind die Verdeutlichung der Dringlichkeit und Skizzierung von Vorschlägen zur Etablierung eines effizienten, anpassungsfähigen und reaktionsbereiten GES-Netzwerkes unter Berücksichtigung von externen Rahmenbedingungen und internen Standards. Die erarbeiteten Vorschläge basieren auf der Grundlage globaler und länderspezifischer Best Practices und Strategiepapiere. Zu den konkreten nächsten Schritten zur Realisierung einer integrierten GES zählen die Ermöglichung der Verknüpfung epidemiologischer Daten mit Genomdaten der Erreger, die gemeinsame und koordinierte Nutzung von vorhandenen Ressourcen, die Nutzbarmachung der so gewonnenen Surveillance-Daten für relevante Entscheidungstragende, den Öffentlichen Gesundheitsdienst und die wissenschaftliche Gemeinschaft sowie die Einbindung aller Stakeholder. Der Aufbau eines GES-Netzwerkes ist essentiell für die kontinuierliche, stabile, aktive Überwachung des Infektionsgeschehens in Deutschland sowohl während pandemischer Phasen als auch außerhalb dieser.

**Zusatzmaterial online:**

Zusätzliche Informationen sind in der Online-Version dieses Artikels (10.1007/s00103-023-03680-w) enthalten.

## Abstract (en)

The SARS-CoV‑2 pandemic has shown a deficit of essential epidemiological infrastructure, especially with regard to genomic pathogen surveillance in Germany. In order to prepare for future pandemics, the authors consider it urgently necessary to remedy this existing deficit by establishing an efficient infrastructure for genomic pathogen surveillance. Such a network can build on structures, processes, and interactions that have already been initiated regionally and further optimize them. It will be able to respond to current and future challenges with a high degree of adaptability.

The aim of this paper is to address the urgency and to outline proposed measures for establishing an efficient, adaptable, and responsive genomic pathogen surveillance network, taking into account external framework conditions and internal standards. The proposed measures are based on global and country-specific best practices and strategy papers. Specific next steps to achieve an integrated genomic pathogen surveillance include linking epidemiological data with pathogen genomic data; sharing and coordinating existing resources; making surveillance data available to relevant decision-makers, the public health service, and the scientific community; and engaging all stakeholders. The establishment of a genomic pathogen surveillance network is essential for the continuous, stable, active surveillance of the infection situation in Germany, both during pandemic phases and beyond.

## Hintergrund

Die SARS-CoV-2-Pandemie hat ein Defizit an essentieller infektionsepidemiologischer Infrastruktur in Deutschland aufgezeigt. Insbesondere sind die infrastrukturellen Voraussetzungen unzureichend, um die Verbreitung und Evolution von Infektionserregern mit Methoden der modernen Genom-Sequenzierung, der sogenannten Genomischen Erreger-Surveillance^1^ (GES; siehe Infobox), zu messen und zu überwachen [1]. Zur Vorbereitung auf zukünftige pandemische Notlagen sehen wir es als dringend erforderlich an, dieses im Vergleich zu anderen Ländern bestehende Defizit durch den Aufbau einer leistungsfähigen Infrastruktur für GES zu beheben. Auch aktuelle und sich verschärfende globale infektionsmedizinische und hygienische Herausforderungen wie die Verbreitung multiresistenter Bakterien bedürfen dringend einer solchen Infrastruktur, um die heutigen Möglichkeiten zur Verhinderung der Ausbreitung von Infektionserregern zur Entfaltung zu bringen [2].

Die Verfassenden dieses Dokuments sind Expert*innen aus „GenSurV“ (Genomic Pathogen Surveillance and Translational Research), einem Teilprojekt des Netzwerks Universitätsmedizin (NUM), aus dem Robert Koch-Institut (RKI) sowie aus weiteren Institutionen und Organisationen der Bioinformatik, Genomik, Mikrobiologie, Virologie, Infektiologie, Hygiene und Infektionsprävention, Epidemiologie und dem Öffentlichen Gesundheitsdienst (ÖGD), inklusive Leiter*innen von Referenz- und Konsiliarlaboratorien^2^. Wir empfehlen dringend den Aufbau eines koordinierten, kollaborativ angelegten GES-Netzwerks mit hoher Effizienz, Anpassungsfähigkeit und Reaktionsbereitschaft in Deutschland. Wir halten es für unverzichtbar, eine derartige Struktur zu schaffen. Sie ist für die kontinuierliche, stabile, aktive Überwachung des Infektionsgeschehens in Deutschland sowohl während pandemischer Phasen als auch außerhalb dieser essentiell. Voraussetzungen für ein solches Netzwerk umfassen die rechtliche Grundlage, insbesondere die datenschutzrechtliche, sowie die technische, finanzielle, personelle und fachliche Nachhaltigkeit.

In diesem Positionspapier werden zunächst die möglichen Strukturen und Strategien einer GES in Deutschland dargestellt. Die Bedeutung der GES im Kontext der SARS-CoV-2-Pandemie und darüber hinaus wird erläutert. Es werden Vorschläge für konkrete Schritte zur Etablierung der GES gemacht sowie Herausforderungen und offene Fragen benannt. Abschließend werden die für den Erfolg der GES notwendigen externen Rahmenbedingungen und internen Standards dargestellt.

## Struktur und Strategie für Genomische Erreger-Surveillance in Deutschland

Ein GES-Netzwerk kann auf Basis bereits regional initiierter Strukturen, Prozesse und Interaktionen diese optimieren und zukünftig mit einer hohen Anpassungsfähigkeit auf aktuelle und zukünftige Herausforderungen reagieren. Darüber hinaus schafft es eine internationale Anschlussfähigkeit durch institutionell (RKI) und personell (Universitätskliniken) bereits bestehende sowie neu zu etablierende Vernetzungen (mit diagnostischen Laboren, Referenz- und Konsiliarlaboratorien, außeruniversitären Einrichtungen, nichtuniversitären Kliniken, Landesgesundheitsbehörden, ÖGD). Die notwendige Struktur umfasst folgende Elemente:

- ein Netzwerk von Laboren zur Isolierung, Identifikation und Sammlung von Erregern,
- Labore mit der Fähigkeit zur Sequenzierung und phänotypischen Charakterisierung gesammelter Erreger-Isolate,
- eine Daten-Infrastruktur zur Sammlung und bioinformatischen Analyse der Genomdaten,
- infektionspräventive Expertise zur Verknüpfung dieser Informationen mit denen zu Person, Ort und Zeit sowie
- eine oder mehrere Stellen zur gebündelten Auswertung mit entsprechenden Schnittstellen zu politischen Entscheidungsträger*innen.

Durch einen modularen Aufbau sind eine schnelle Anpassung und Skalierbarkeit auf weitere Public-Health-relevante Erreger, wie z. B. multiresistente Bakterien oder Influenzaviren, gegeben. Grundsätzlich sind die genannten Einzelelemente in Deutschland vorhanden. Es mangelt jedoch an Ressourcen, einem entsprechenden Auftrag, korrespondierender Ausstattung sowie an Legitimation und Koordination [3].

Die Weltgesundheitsorganisation (WHO) hat eine globale Strategie zur genomischen Surveillance von Pathogenen mit pan- und epidemischem Potenzial formuliert [4]. Beispiele für erfolgreiche Programme sind z. B. im Vereinigten Königreich, Dänemark, Australien oder Neuseeland zu finden [1].

Wir schlagen deshalb vor, basierend auf dieser Strategie und den erfolgreichen Programmen anderer Länder, ein GES-Netzwerk auch in Deutschland zügig und konsequent aufzubauen und zu fördern. Dieses soll in der Lage sein, das ständige Infektionsgeschehen auch außerhalb ungewöhnlicher Vorkommnisse zu überwachen (z. B. bakterielle Antibiotikaresistenz, lebensmittelassoziierte Infektionen, saisonale Virusinfektionen) mit der Möglichkeit einer schnellen Anpassung an epi- oder pandemische Infektionsgeschehen. Die modernen Methoden der Analyse von Erregergenomen lassen schnelle Rückschlüsse auf die Art der Ausbreitung und die Entstehung von ggf. besorgniserregenden Varianten von Infektionserregern zu [5–8].

Hierdurch wird die wissenschaftliche Grundlage geschaffen, um fundierte Empfehlungen für politische Entscheidungen zu versorgungsbezogenen und gesellschaftlichen Herausforderungen und deren Bewältigung zur Verfügung stellen zu können. Konkret ermöglicht sie eine verbesserte Steuerung der Maßnahmen zur Vermeidung von Infektionen und Todesfällen (z. B. Impfstrategie; Public-Health-Maßnahmen) durch die Gewinnung von Erkenntnissen zur Entstehung, Verbreitung, Evolution und Infektionsprävention von Krankheitserregern in Deutschland [9].

Bezogen auf die SARS-CoV-2-Pandemie stellt die kombinierte Nutzung von genomischen Erregerinformationen mit Angaben zu Person, Ort und Zeit zur verbesserten Charakterisierung von SARS-CoV-2-Übertragungsketten in der Bevölkerung ein großes, bisher wenig genutztes Potenzial zur Verbesserung des Pandemiemanagements dar. Die Integration dieser Informationen in das Kontaktmanagement der lokalen Gesundheitsbehörden könnte somit einen erheblichen gesellschaftlichen Nutzen schaffen, sofern organisatorische und technologische Herausforderungen bewältigt werden [10, 11].

## Notwendigkeit und Bedeutung des Aufbaus der Genomischen Erreger-Surveillance in Deutschland für pandemische und nicht-pandemische Zeiten

Die Pandemie hat die Bedeutung der GES und einer engmaschigen Überwachung der vorherrschenden Virusvariante für individuelle Therapie- und gesundheitspolitische Entscheidungen eindrücklich vor Augen geführt [12, 13]. Die GES erlaubt eine Echtzeitabbildung des Infektionsgeschehens auf Bevölkerungsebene und die Erkennung von Veränderungen des Erregers. Maßnahmen zur Eindämmung der Pandemie konnten darauf basierend z. B. an Erkrankungsschwere und Ansteckungspotenzial der vorherrschenden Variante angepasst werden. Die Behandlung individueller Patient*innen profitierte von der GES u. a. durch die verbesserte Auswahl passender therapeutischer Antikörper, da viele therapeutische Antikörper nicht universell, sondern nur bei bestimmten Virusvarianten wirken [5–8].

Es ist wichtig zu betonen, dass aktuell unter den besonderen Bedingungen der SARS-CoV-2-Pandemie durch den niedrigschwelligen Testzugang, die Refinanzierung der Test- und Sequenzierkosten und die gesetzlichen Rahmenbedingungen zur verpflichtenden Datenübermittlung wichtige Fortschritte für eine GES in Deutschland erzielt wurden (siehe Onlinematerial II). Um diese Erfolge in ein kontinuierliches nachhaltiges System bestehend aus personeller, prozessualer und technischer Infrastruktur zu überführen, bedarf es Anpassungen auch für andere Infektionserreger. Hierbei ist u. a. die genomische Surveillance von Antibiotikaresistenzdeterminanten von wachsender Bedeutung [14], da die bislang gegen multiresistente, gramnegative Bakterien zugelassenen und in Entwicklung befindlichen Antibiotika nicht bei allen Resistenzdeterminanten wirken. Die durch die GES erfasste Verteilung von Resistenzgenen (z. B. Art der Carbapenemase) ermöglicht eine Abschätzung der regionalen Wirksamkeit einer neuen Substanz. Dabei ist es die Aufgabe der Wissenschaft, durch umfassende Zusammenstellung der möglichen Erkenntnisse mit zielgerichteten Empfehlungen optimale (gesundheits-)politische Entscheidungen zu ermöglichen und zur Verbesserung der Gesundheitsversorgung beizutragen. Ein wesentliches Modul in diesem Diskurs sind die umfassende Gewinnung und Aufbereitung genomischer Surveillance-Daten, welche zurzeit in Deutschland fehlt.

Zur Vorbereitung auf zukünftige pandemische Notlagen sehen wir es als dringend erforderlich an, diese Defizite durch den Aufbau eines leistungsfähigen Netzwerks für GES zu beheben. Auch bereits stattfindende globale infektionsmedizinische und hygienische Herausforderungen bedürfen eines solchen Netzwerks. In nicht-pandemischen Zeiten wird es z. B. auch entscheidend dazu beitragen, die Verbreitung multiresistenter Krankenhauskeime effektiver einzudämmen. Neben der Verbreitung multiresistenter Erreger werden zunehmend Ausbrüche detektiert, bei denen sich Antibiotikaresistenzdeterminanten über Speziesgrenzen hinweg verbreiten und sogenannte „Multispeziesausbrüche“ verursachen. Auch diese Ausbrüche können nur mit einer funktionierenden GES erkannt werden.

Die Entwicklung eines GES-Netzwerks mit hoher Flexibilität, Adaptionsfähigkeit, internationaler Anschlussfähigkeit und Skalierbarkeit hat eine hohe Priorität. Auf Grundlage der in Deutschland existierenden Strukturen wird dies ermöglicht durch die Erschließung und Zusammenführung der komplementären Expertise der Akteur*innen, die Etablierung einer effizienten Koordinierung, Zusammenarbeit und Aufgabenteilung sowie die gemeinsame Nutzung von etablierten und zu entwickelnden Infrastrukturen. Eine auf dem Netzwerkansatz basierende Implementierung der GES in Deutschland folgt ähnlich strukturierten Programmen in z. B. dem Vereinigten Königreich, Dänemark, Australien oder Neuseeland.

So entsteht ein Netzwerk mit koordinierter Vorgehensweise zur Proben- und Datengewinnung, welche für die Klärung flexibler, definierter Fragestellungen herangezogen werden kann, die z. B. über die in der Coronavirus-Surveillanceverordnung – CorSurV – (im Falle von SARS-CoV-2) benannten Pflichtdaten hinausgehen. Die gewonnenen Daten ermöglichen interventionsrelevante Aussagen über Herkunft, Ansteckungsquellen und krankmachende Eigenschaften (Virulenz), insbesondere bei Bakterien mit Unempfindlichkeit gegenüber Antiinfektiva (antimikrobielle Resistenz – AMR), sowie über Ansteckungsfähigkeit und Übertragungsdichte von Infektionserregern. Dies ist besonders wichtig bei solchen Infektionserregern, die sich durch ihre absehbare Public-Health-Relevanz, d. h. durch hohe Verbreitungsfähigkeit, hohe Erkrankungslast und unkalkulierbares wirtschaftliches Schadenspotenzial, von anderen Mikroorganismen unterscheiden.

Das GES-Netzwerk soll außerhalb ungewöhnlicher Infektionsgeschehen relevante Verschiebungen in der Epidemiologie von Infektionserregern erkennen, als Grundlage für die schnelle Reaktionsfähigkeit bei epi- oder pandemischen Geschehen genutzt werden und Einfluss auf versorgungsbezogene und gesellschaftliche Fragestellungen haben. Die GES ist dabei schnell auf weitere Erreger von unbestrittener Public-Health-Relevanz skalierbar, da die zugrunde liegende Technologie der Genomsequenzanalyse generisch nutzbar ist. Sie kann dadurch in Deutschland weiter an Tragweite gewinnen und auch im internationalen Vergleich konkurrenzfähig werden.

## Ziel einer Genomischen Erreger-Surveillance in Deutschland, konkrete Schritte und Herausforderungen

Optimierte und gezielte Steuerung der Maßnahmen zur Vermeidung von Infektionen und Todesfällen (z. B. Impfstrategie) durch Gewinnung von Erkenntnissen zur Verbreitung, Evolution und Infektionsprävention von bakteriellen und viralen Krankheitserregern in Deutschland.

Vorschläge für konkrete Schritte:

- Aufbau eines nationalen Netzwerks für die GES von Infektionserregern, um deren Entstehung, Verbreitungsdynamik, Diversität und Evolution von neuen klonalen Linien durch vergleichende Ganzgenom-Sequenzanalysen zu erfassen.
  Dabei wird ein integrierter Surveillance-Ansatz verfolgt, bei dem ein Zusammenfügen von epidemiologischen Kerndaten (Person, Ort, Zeit) und Genomdaten der Erreger erfolgt, welcher die Voraussetzung für die Nutzung als Steuerungsparameter und einen Erkenntnisgewinn darstellt.
- Erschließung und Zusammenführung der komplementären Techniken (z. B. Next und Third Generation Sequencing) und Expertisen der Akteur*innen, Etablierung einer effizienten Koordinierung, Zusammenarbeit und Aufgabenteilung sowie gemeinsame Nutzung von Analyseplattformen für Sequenzdaten und die Weiterentwicklung von (Forschungs‑)Infrastrukturen.
  Die Konzeptionierung als Netzwerk ist erforderlich, um alle relevanten Expertisen dezentral, agil und adaptiv nutzen zu können. Dabei liegt der primäre Fokus von Surveillance auf einem Verständnis der Ausbreitung, Übertragung und Entwicklung der Infektionserreger als Grundlage für angemessene Entscheidungsfindungen in Versorgung und Infektionsprävention. Darüber hinaus ermöglicht es die Konzeptionierung als Netzwerk, gemeinschaftlich auch wissenschaftliche Fragestellungen anhand dieser Daten zu beantworten.
- Nutzbarmachung der so gewonnenen Surveillance-Daten für relevante Entscheidungsträger*innen, den ÖGD und die wissenschaftliche Gemeinschaft.
  Dabei sollten die komplementären Expertisen und Infrastrukturen der relevanten Akteur*innen, d. h. des RKI, der Universitätskliniken, der diagnostischen Labore, der Nationalen Referenzzentren und Konsiliarlaboratorien sowie außeruniversitärer Einrichtungen, nichtuniversitärer Kliniken, Landesgesundheitsbehörden und ÖGD, gebündelt und genutzt werden, um Fragestellungen der GES einschließlich Erregerpriorisierung (siehe Onlinematerial III) und Beprobungsstrategien adaptiv auszuarbeiten und anzuwenden.
- Einbindung aller Akteur*innen: Das Netzwerk der Verfassenden ist *a priori* kollaborativ initiiert sowie abgestimmt und ging aus der Zusammenarbeit des Projekts GenSurV und des RKI hervor. Weitere Expert*innen aus den Bereichen Bioinformatik, Epidemiologie, Genomik, Hygiene, Infektionsprävention und -kontrolle, Infektiologie, Mikrobiologie und Virologie sowie Vertreter*innen bestehender Infrastrukturen wie Referenz- und Konsiliarlaboratorien als auch Landesgesundheitsbehörden wurden als gleichberechtigtes Expert*innenpanel integriert. Die Initiierung des Netzwerks erfolgte auf einem Workshop am 06. und 07.07.2022 in Berlin. Es soll zukünftig kollaborativ weitergeführt werden und eine Teilnahme soll interessierten Parteien offenstehen, um Wahrnehmung und Aussagekraft der GES durch eine möglichst breite Teilnehmendenbasis zu stärken.

Es ergeben sich folgende Herausforderungen und offene Fragen:

- Welche Voraussetzungen müssen für eine erfolgreiche GES vorliegen?
- Welche personellen, prozessualen und technischen Infrastrukturkomponenten sind notwendig?
- Wie gelingt es, eine solche Infrastruktur *a priori* international anschlussfähig, skalierbar und nachhaltig zu etablieren?
- Für welche Infektionserreger sollte eine GES durchgeführt werden, wie sind diese zu priorisieren?
- Welche Probengewinnungsstrategien sind für (i.) welche Fragestellung und (ii.) welche Infektionserreger am besten geeignet?
- Welche Rolle hat dieses als adaptives System angedachte Netzwerk in Pandemiezeiten und in Nicht-Pandemiezeiten?

Die hier skizzierte Strategie folgt der bereits genannten globalen Strategie der WHO zur genomischen Surveillance von Pathogenen mit pan- und epidemischem Potenzial [4] und adaptiert sowie konkretisiert die dort genannten Ziele, Vorgehensweisen und Fragestellungen für Deutschland (siehe Onlinematerial IV).

## Externe Rahmenbedingungen und interne Standards für eine erfolgreiche Genomische Erreger-Surveillance

Um die Umsetzung einer funktionierenden GES in Deutschland sicherzustellen, sind folgende Voraussetzungen erforderlich:

- Rechtliche Grundlagen
- Nachhaltigkeit
- Grundsätze der Datengenerierung, Qualitätskontrolle und Datenanalyse
- Grundlagen der surveillancebasierten Entscheidungsfindung und Risikokommunikation
- Einbindung aller Akteur*innen
- Governance
- Kommunikation
- Agilität und Skalierbarkeit

Die Erfolgskriterien teilen sich auf in durch *von außen* zu schaffende Rahmenbedingungen, durch *netzwerkintern* zu regelnde Voraussetzungen sowie durch *in Abstimmung* umzusetzende Kriterien.

### (a) Rechtliche Grundlagen.

Für eine systematische GES werden klare rechtliche Rahmenbedingungen benötigt. Angelehnt an die Coronavirus-Surveillanceverordnung (CorSurV) sollten die Grundlagen geschaffen werden, die eine GES zur Verhütung und Bekämpfung von Infektionskrankheiten beim Menschen erlauben/ermöglichen (z. B. Infektionsschutzgesetz). Insbesondere sollte eine explizite rechtliche Grundlage für die Generierung, Sammlung, Verarbeitung und pseudonymisierte Veröffentlichung von Erreger-Sequenzierungsdaten zum Zwecke der GES geschaffen werden. Diese soll auf angemessene Weise die Aspekte des Datenschutzes mit der Möglichkeit einer besseren Prävention und Behandlung lebensbedrohlicher Infektionen vereinen, die durch ein modernes GES-System geboten wird.

Um zu verhindern, dass Unklarheiten in Hinblick auf datenschutzrechtliche Regelungen zu signifikanten Verzögerungen bei der Sammlung dringend benötigter Daten führen, wie es in der SARS-CoV-2-Pandemie häufig der Fall war, sollten folgende Aspekte bei der Schaffung rechtlicher Grundlagen unbedingt und explizit Berücksichtigung finden:

1. die zentrale Speicherung, Analyse und Veröffentlichung von pseudonymisierten Erreger-Sequenzierungsdaten und Metadaten, die eine räumliche und zeitliche Zuordnung der gespeicherten Sequenzierungsdaten erlauben;
2. der Zugriff auf die unter Punkt 1. genannten Daten durch die wissenschaftliche Gemeinschaft, analog zu internationalen und durch die WHO empfohlenen Standards („early, safe, transparent and rapid sharing of samples and genetic sequence data of pathogens of pandemic and epidemic, or other high-risk, potential“ [15]);
3. die Zusammenführung von Erreger-Sequenzierungsdaten mit weiteren personenbezogenen Daten durch die verantwortlichen Gesundheitsbehörden zum Zwecke einer Analyse und gezielten Unterbrechung von Infektionsketten;
4. die Standards, die bei diesen Prozessen in Bezug auf die Gewährleistung eines hinreichenden individuellen Datenschutzes zu beachten sind.

### (b) Nachhaltigkeit.

Es ist essentiell, (i.) eine Kontinuität der Funktionalität einer Kerninfrastruktur durch Bearbeitung von Fragestellungen hoher nationaler Relevanz zu gewährleisten sowie (ii.) durch regelhafte Übungsfälle für potenzielle Lagen deren Expansion zu prüfen, um (iii.) schnell, qualitätsgesichert und abgestimmt in epidemischen oder pandemischen Lagen jederzeit hochskalieren zu können. Grundvoraussetzung ist eine dauerhaft gesicherte Finanzierung personeller, prozessualer, technischer Infrastrukturen sowie der Sequenzierung an sich, um einen nachhaltigen, qualitätsgesicherten Aufbau und Betrieb des GES-Netzwerks zu garantieren.

Auf der anderen Seite ist eine effiziente Verwendung der Ressourcen notwendig. Darunter fallen die optimierte Etablierung und Nutzung von Strukturen, Prozessen und Personen, wie z. B. die Entwicklung von möglichst effizienten Beprobungsstrategien sowie aller vor- und nachgeschalteten Prozesse, aber auch die Auswahl und Reihenfolge der aufgenommenen Infektionserreger. Dazu bedarf es einer qualifizierten und koordinierten Bewertung, welche die Interessen der Beteiligten berücksichtigt.

### (c) Grundsätze der Datengenerierung, Qualitätskontrolle und Datenanalyse.

Validität und Qualität der generierten Sequenzierungs- und gesammelten Metadaten sind wichtige Erfolgsfaktoren für die GES. Hier muss insbesondere sichergestellt werden, dass (i.) Datensätze in Bezug auf Person, Ort und Zeit korrekt zugeordnet werden, (ii.) stringente Qualitätskriterien bei der Generierung der Rohdaten angewandt werden, um z. B. die Verwechslung oder Vermischung von Proben zu verhindern, (iii.) validierte Algorithmen und Pipelines für die Datenanalyse zum Einsatz kommen. Dafür kann in vielen Bereichen auf etablierte Prozesse und „Best Practices“ zurückgegriffen werden, so z. B. im Bereich der primären Datenerzeugung (Good Laboratory Practice, ISO-QM-Normen), im Bereich der bioinformatischen Softwareentwicklung (Versionierung, Containerisierung, Reproduzierbarkeit) und im Bereich der Analyse und Dateninterpretation (Bestätigung wichtiger Analyseergebnisse durch mehrere unabhängige Analysemethoden und Forschungsgruppen). Um das Potenzial der GES vollumfänglich zu nutzen, muss – unter Berücksichtigung datenschutzrechtlicher Aspekte – eine Verknüpfung relevanter Metadaten mit den Sequenzierungsdaten ermöglicht werden.

### (d) Grundlagen der surveillancebasierten Entscheidungsfindung und Risikokommunikation.

Um den Mehrwert der durch GES gewonnenen Daten in Informationen und Erkenntnisse sowie letztlich Entscheidungsfindungen zu überführen, bedarf es klarer kollaborativ konstituierter Standards. Die SARS-CoV-2-Pandemie hat deutlich aufgezeigt, dass die Nutzung und Nutzbarmachung von Erkenntnissen sowohl für die Gesundheitsversorgung als auch für gesundheitspolitische Entscheidungen hoch relevant sind. Um diese Prozesse zu erleichtern und sowohl die Optionen als auch die Grenzen vorliegender Daten besser sichtbar zu machen, sollten diesbezüglich abgestimmte Standards als Orientierungsrahmen geschaffen werden. Darüber hinaus sollten für eine schnelle Einschätzung komplexer Sachverhalte eine Logistik und Infrastruktur errichtet werden, die ein Meinungsbild von Expert*innen schnell, repräsentativ und zuverlässig einzuholen vermag. So kann die Beratung der gesellschaftlichen und politischen Entscheidungsträger*innen optimiert werden.

### (e) Einbindung aller Akteur*innen.

Die GES sollte *a priori* kollaborativ angelegt sein. GenSurV und das RKI haben den Prozess initiiert, um den Mehrwert der Zusammenarbeit zu nutzen und Parallelentwicklungen zu vermeiden. Darüber hinaus ist die Wahrung der Interessen aller Beteiligten erforderlich. Es muss sichergestellt sein, dass die Daten- und Materialliefernden zu jedem Zeitpunkt der Kollaboration Besitzende ihrer Daten sind und als solche aufgeführt werden. Eine *Open-Data*-Strategie sollte verfolgt und die wissenschaftliche Gemeinschaft niedrigschwellig eingebunden werden. Durch Anwendung der FAIR-Prinzipien („*F*indable, *A*ccessible, *I*nteroperable, and *R*e‑usable“) kann dafür eine Grundlage geschaffen werden. International erfolgreiche Surveillance-Programme zeigen zudem den Wert einer öffentlichen Bereitstellung von Erreger-Genomdaten.

Um eine möglichst hohe Akzeptanz unter allen Beteiligten (Universitätskliniken, RKI, diagnostische Labore, Nationale Referenzzentren und Konsiliarlaboratorien, außeruniversitäre Einrichtungen, nichtuniversitäre Kliniken, Landesgesundheitsbehörden, ÖGD) zu erreichen, sollten die Datenlieferung und -verwendung möglichst niedrigschwellig und zeitnah, jedoch ohne Qualitätsverluste gewährleistet sein.

### (f) Governance.

Um mittel- und langfristig die Funktion der GES in Deutschland sicherzustellen, sind die Prozesse und Rollen der diversen Netzwerkpartner*innen klar festzulegen. Möglichst schlanke Entscheidungsgremien und -prozesse müssen definiert sein. Beratende Gremien sollten unter bestmöglicher Nutzung bestehender Strukturen initiiert werden. Um eine Stabilität zu gewährleisten, muss eine Kontinuität hinsichtlich der beteiligten Personen sichergestellt werden. Darüber hinaus sollte eine Offenheit bestehen, um Innovations- und Modernisierungsüberlegungen Rechnung zu tragen. Auf eine angemessene Repräsentanz verschiedener Gruppen muss geachtet werden.

### (g) Kommunikation.

Interne und externe Kommunikation sind von zentraler Bedeutung für die erfolgreiche Umsetzung. Eine offene Kommunikation zwischen allen Beteiligten ist essentiell für die Weiterentwicklung des Netzwerks, da sie, angelehnt an den PDCA-Zyklus (*P*lan-*D*o-*C*heck-*A*ct), Möglichkeiten zur Ideenentwicklung, zum fachlichen Austausch sowie zu Feedback und Überarbeitung gibt. Der resultierende Mehrwert, den die Agierenden aus der Zusammenarbeit ziehen, muss erkennbar sein und kommuniziert werden. Darüber hinaus muss das Netzwerk diesen Mehrwert und die Inhalte der Arbeit nach außen kommunizieren, aber ebenso für Anregungen, Fragen und Aufträge von extern zur Verfügung stehen und geeignete Kommunikationskanäle etablieren und erhalten.

### (h) Agilität und Skalierbarkeit.

Eine Schlüsselaufgabe für das GES-Netzwerk besteht in der Etablierung aller erforderlichen strukturellen, personellen, prozessualen und technischen Infrastrukturkomponenten dergestalt, dass diese:

I. in einem *vorhersagbaren kontinuierlichen Aktivitätszustand*
  (i.) infektionsepidemiologisch relevante Erreger und Erkrankungen, wie z. B. multiresistente Krankenhauserregern adressieren und
  (ii.) in geeigneten Intervallen durch Stresstests die Skalierbarkeit für II. üben.
II. in einem *gesteigerten Aktivitätszustand* sowohl den dann neuen umfangreichen Aufgabenstellungen gerecht werden können, ohne ggf. relevante Routinetätigkeiten komplett zu vernachlässigen. Dabei kann sich die erhöhte Aktivität
  (iii.) vorhersehbar in einem moderaten Ausmaß (z. B. jährliche saisonale Influenza-Aktivität) bewegen oder
  (iv.) nicht vorhersehbar im Kontext einer Pandemie auf einem sehr hohen Aktivitätsniveau liegen.

Um diese neue Funktionalität zu etablieren, bedarf es eines profunden Konzeptes, aber auch einer soliden Basis mittels der unter I. benannten Aufgaben.

## Fazit

Eine solche abgestimmte, dezentrale und alle Expertisen umfassende Struktur und Strategie für GES ist für die kontinuierliche, stabile, aktive Überwachung des Infektionsgeschehens in Deutschland – sowohl während pandemischer Phasen als auch außerhalb dieser – für wichtige Aufgaben wie das Management multiresistenter Erreger und nosokomialer Infektionen essentiell. Nur durch ein solches Netzwerk kann die Realisierung des Potenzials der GES zur Verbesserung der Versorgungssicherheit und -qualität in Deutschland sichergestellt werden.

### Infobox Genomische Erreger-Surveillance (GES) – Verständnis verschiedener Termini aus Sicht der Autor*innen

*Surveillance *ist das wichtigste Instrument der überwachenden Epidemiologie. Sie dient der Wahrnehmung, Quantifizierung und Lokalisierung von Gesundheitsereignissen und deren Determinanten. Voraussetzung dafür sind eine systematische, strukturierte Erfassung sowie die zielgruppenspezifische Kommunikation mit relevanten Stakeholdern. Die Beurteilung von Gesundheitsbedrohungen und die eventuell notwendige Einleitung von Kontrollmaßnahmen können so gewährleistet werden. Dabei handelt es sich um einen kontinuierlichen, adaptiven Prozess.

*Genomische Erreger-Phylogenie* nutzt die Verfügbarkeit von Genomsequenzen individueller Infektionserreger (die Gesamtheit der genetischen Information), um die stammesgeschichtlichen Beziehungen (Abstammung) aller einzelnen Erregerisolate untereinander abzubilden. Stehen darüber hinaus Informationen über den Zeitpunkt und den Ort (Geolokalisation) der Entnahme zur Verfügung, ist es möglich, die Verbreitung und Übertragungsdynamik von Infektionen über Raum und Zeit in der Bevölkerung nachzuvollziehen (Geophylogenie).

Die *Genomische Erreger-Surveillance (GES) *ist die Verknüpfung der infektionsepidemiologischen Daten von Patient*innen, z. B. Meldedaten und Genomsequenzierungsdaten, sowie deren schnelle systematische Analyse zur Ausbruchsdetektion für gezielteres Handeln und für die Bestimmung epidemiologischer Trends.

## Supplementary Information





## Footnotes

1 Weiterführende Informationen zum Begriffsverständnis und zum Potenzial der GES sind im Onlinematerial I nachzulesen.

2 https://www.rki.de/DE/Content/Infekt/NRZ/nrz_node.html.
