# Supplementary material for: Establishment of genomic pathogen surveillance to strengthen pandemic preparedness and infection prevention in Germany
Source: Bundesgesundheitsblatt Gesundheitsforschung Gesundheitsschutz. 2023 Feb 22;66(4):443–9. [Article in German] doi: 10.1007/s00103-023-03680-w (PMC9945818; doi:10.1007/s00103-023-03680-w)
Supplement: Supplementary file 1 [file 103_2023_3680_MOESM1_ESM.pdf]

Zusätzliches Onlinematerial zum Artikel:

Etablierung der Genomischen Erreger-Surveillance zur Stärkung des Pandemie- und Infektionsschutzes in Deutschland

Simone Scheithauer, Alexander Dilthey, Anna Bludau, Sandra Ciesek, Victor Corman, Tjibbe Donker, Tim Eckmanns, Richard Egelkamp, Hajo Grundmann, Georg Häcker, Martin Kaase, Berit Lange, Alexander Mellmann, Martin Mielke, Mathias Pletz, Bernd Salzberger, Andrea Thürmer, Andreas Widmer, Lothar Wieler, Thorsten Wolff, Sören Gatermann, Torsten Semmler

Verzeichnis

I. Was ist und kann Genomische Erreger-Surveillance?
II. Nutzung von Genomischer Erreger-Surveillance in Deutschland - Aktueller Stand
III. Erregerpriorisierung: Auswahl der Infektionserreger mit Public-Health-Relevanz
IV. Globales Strategiepapier zur Genomischen Erreger-Surveillance der Weltgesundheitsorganisation (WHO, März 2022)

I. Was ist und kann Genomische Erreger-Surveillance?

Um der Vision einer optimierten Steuerung in Pandemie- und Krisensituationen mit Unterstützung durch Genomische Erreger-Surveillance (GES) näherzukommen, mit dem Ziel, schwere Infektionen und Todesfälle durch virale und (auch multiresistente) bakterielle Infektionserreger zu reduzieren und effektive Maßnahmen fokussiert einzusetzen, bedarf es eines gemeinsamen Verständnisses der Genomischen Surveillance.

Obgleich regelhaft verwendet, können einzelne Termini doch unterschiedlich z. B. hinsichtlich Bedeutung, Umfang und Zielsetzung genutzt werden, sodass wir, um Missverständnissen vorzubeugen, die wichtigsten Termini und Hintergrundinformationen konzis aus Sicht der Verfassenden mit Blick auf GES in der Infobox I innerhalb des Positionspapier dargestellt haben.

Die GES ist die Voraussetzung für eine Früherkennung evolutionärer Varianten mit erhöhtem Risikopotenzial wie veränderter Pathogenität, Übertragungsfähigkeit, Wirtsspektrum (Ältere, Kinder, Jugendliche), Immunevasion und Antibiotikaresistenz und somit ein wichtiges Instrument zur Verbesserung von Versorgungssicherheit. Dabei stellt die GES ein additives und synergistisches Element der klassischen infektionsepidemiologischen Surveillance dar[1].

Weitere wichtige Potenziale liegen im Einsatz der Surveillance zum besseren Verständnis von Infektionsketten. Diese Informationen können genutzt werden, um beispielsweise in Kontexten wie Altenheimen und Krankenhäusern Hygienemaßnahmen zu verbessern. Letztlich liefert die GES auch Daten, die für die wissenschaftliche Auseinandersetzung mit neuen und bestehenden Krankheitserregern von entscheidender Bedeutung sind – so z. B. im Bereich der Pathogen-Evolutions- und Populationsgenetikforschung. Internationale Beispiele für eine erfolgreich etablierte Genomische Erreger-Surveillance zeigen bereits deutlich deren Mehrwert für die Bereiche Forschung, Datenintegration mit Public-Health-Relevanz und auch Mitarbeitertraining und -qualifikation. Dem „COVID-19 Genomics UK Consortium“ und auch dem „Danish Covid-19 Genome Consortium“ ist es unmittelbar nach Beginn der SARS-CoV-

[1] Weltgesundheitsorganisation (2021) Genomic sequencing of SARS-CoV-2: A guide to implementation for maximum impact on public health. http://apps.who.int/iris/bitstream/handle/10665/338480/9789240018440-eng.pdf?sequence=1&isAllowed=y#page34. Zugegriffen am 06. Januar 2023

2-Pandemie gelungen, ein landesweites nachhaltiges Netzwerk von Genomsequenzierung, -analyse und Datenintegration aufzubauen, das den Einrichtungen des öffentlichen Gesundheitsdienstes wichtige Daten für Entscheidungen zur Verfügung stellen kann. In beiden Ländern werden die etablierten Strukturen und Prozesse nun genutzt, um die GES auf weitere Public-Health-relevante Mikroorganismen auszuweiten.

II. Nutzung von Genomischer Erreger-Surveillance in Deutschland - Aktueller Stand

In mehreren Ländern, wie auch Deutschland, wird die Gesamtgenomsequenzierung von Infektionserregern an Universitätskliniken und Referenzzentren, Konsiliarlaboratorien, Landesgesundheitsbehörden und im ÖGD zunehmend als entscheidende Voraussetzung für wirksame und gezielte Interventionen sowohl bei der klinisch-individuellen Patient*innenversorgung als auch bei der bevölkerungsbezogenen Infektionskontrolle angesehen. Nach der gesetzlichen Grundlage für die SARS-CoV-2-Surveillance in Deutschland (CorSurV, 19.01.2021) und den Empfehlungen des RKI sind bereits zum jetzigen Zeitpunkt zwei grundsätzliche Vorgehensweisen umzusetzen: Neben der derzeit praktizierten sog. Zufallsstichprobe aus allen positiven Laborproben verweist die Testverordnung auch auf die Relevanz von Überwachungsproben (surge sampling) bei Verdacht auf Fallverdichtung oder bei unklaren Infektionsgeschehen.

Der tatsächliche Nutzen der Ganzgenomsequenzierung von Infektionserregern hängt jedoch von einer Reihe von Faktoren ab, u. a. (i.) von geeigneten Beprobungsstrategien, (ii.) von der nachgeschalteten Nutzung der Daten, insbesondere für gezielte epidemiologische Untersuchungen und die Prozesssteuerung innerhalb des ÖGD und der wissenschaftlichen Gemeinschaft, (iii.) von der Verknüpfung der Genomsequenzierung mit der phänotypischen Charakterisierung, (iv.) von einem kollaborativen, abgestimmten und vernetzten Vorgehen, inklusive Standardisierung.

Daher wurde die komplementäre Expertise und Infrastruktur des NUM im Rahmen der Projekte B-FAST (Bundesweites Forschungsnetzwerk angewandte Surveillance und Testung) und GenSurV (seit 2022) sowie des RKI, insbesondere im Rahmen der vom Bundesministerium für Gesundheit geförderten Projekte IMS-NRZ/KL und IMS-RKI zur Etablierung einer erregerübergreifenden integrierten molekularen Surveillance am RKI, zusammengeführt. Gemeinsam wurde eine integrierte Plattform zur genomischen SARS-CoV-2-Surveillance entsprechend der Vorgaben durch die CorSurV aufgebaut. Um alle relevanten Expertisen zu bündeln, wurde zusätzlich eine verschiedene Fachkenntnisse und Rollen umfassende Expert*innengruppe einberufen und so ein Panel zur zielgerichteten Bearbeitung gebildet.

Darüber hinaus gibt es seit Mitte 2021 in Deutschland das Netzwerk „miGenomeSurv"[2], das anhand von dezentral erstellten Ganzgenomsequenzierungsdaten automatisiert die Erregerüberwachung bei sektorübergreifenden Infektionserregern wie z. B. Listerien oder enterohämorrhagischen *E. coli* (EHEC) unterstützt.

III. Erregerpriorisierung: Auswahl der Infektionserreger mit Public-Health-Relevanz

Das Expert*innenpanel ist der Ansicht, dass bei der Skalierung auf weitere Infektionserreger schrittweise vorzugehen ist, um effektiv und effizient den bestmöglichen Mehrwert zu generieren. Da nicht alle Infektionserreger gleichzeitig betrachtet werden können, wurde eine Priorisierung erstellt mit dem Ergebnis, dass zunächst eine Strategie für SARS-CoV-2, Influenza sowie antibiotikaresistente Erreger ausgearbeitet wird. Als Basis wurden bereits im

---

[2] www.miGenomeSurv.org

Vorfeld unabhängig voneinander erstellte Prioritätslisten des RKI sowie von GenSurV herangezogen. Zur weiteren Eingrenzung wurde eine Bewertungsmatrix erstellt, um Objektivität und Transparenz zu gewährleisten. Diese als Entscheidungsgrundlage dienende Bewertungsmatrix umfasste die folgenden Kriterien: Relevanz für individuelle Patient*innen (Morbidität, Mortalität), Übertragungspotenzial, Fallzahl, Potenzial der Vermeidbarkeit, Relevanz für den Krankenhaussektor, Relevanz für die Bevölkerung, entstehende Zusatzkosten für das Gesundheitssystem, entstehende indirekte Kosten, internationale Relevanz, pandemisches Potenzial, Expertise in Deutschland, bereits etablierte Strukturen die integriert werden können und wissenschaftliche Bedeutung.

Als Ergebnis des Workshops vom 06.07. und 07.07.2022 wurden zwei Erregergruppen identifiziert: Antibiotikaresistente Bakterien und Influenza A/B-Viren. Der Einschluss von antibiotikaresistenten Bakterien könnte z. B. durch Carbapenemase-bildende Enterobacterales erfolgen. Eine Erweiterung des Netzwerkes um weitere Infektionserreger wird im Rahmen künftiger Dialoge angestrebt. Eine schnelle Integration weiterer Infektionserreger soll durch eine komponentenbasierte Struktur gewährleistet werden.

IV. Globales Strategiepapier zur Genomischen Erreger-Surveillance der Weltgesundheitsorganisation (WHO, März 2022)

Die WHO verdeutlicht in ihrem globalen Strategiepapier über die genomische Surveillance für Pathogene mit pan- und epidemischem Potenzial (2022 bis 2032)[3] die internationale Bedeutung der (Weiter-)Entwicklung ebendieser genomischen Surveillance für die öffentliche Gesundheit.

Als Ziel dieser Strategie werden die Stärkung und Skalierbarkeit der genomischen Surveillance für Erreger mit pan- und epidemischem Potenzial benannt, um qualitätsgesichert, schnell und angemessen Maßnahmen im Bereich der öffentlichen Gesundheit innerhalb lokaler bis globaler Surveillance-Systeme durchführen zu können. Um dieses übergeordnete Ziel zu erreichen, hat die WHO fünf Teilziele definiert. Jedes Unterziel wird durch eine Reihe strategischer Maßnahmen untermauert.

Die Ziele und Maßnahmen wurden aus nationaler Perspektive gespiegelt, haben Einzug in die Strategie genommen und sollen in der Folge entsprechend der WHO-Empfehlung bearbeitet werden.

<u>(1.) Verbesserung des Zugangs zu Werkzeugen für eine bessere geographische Repräsentativität</u>

Ziel ist es, nicht nur den Zugang zu präzisen und sensitiven Laborsystemen zu erweitern, sondern auch die **computergestützte Infrastruktur auszubauen sowie die richtige Analytik bereitzustellen, um die Interpretation und Kontextualisierung der komplexen Informationen zu ermöglichen.** Dies ist unter der Prämisse der Finanzierbarkeit, Passung und Anwendbarkeit zu verstehen.

[3] Weltgesundheitsorganisation (2022) Global genomic surveillance strategy for pathogens with pandemic and epidemic potential, 2022–2032. https://www.who.int/publications/i/item/9789240046979. Zugegriffen 06. Januar 2023

(2.) Stärkung der personellen Ressourcen zur Gewährleistung von Schnelligkeit, Skalierung und Qualität

Die personellen Kapazitäten sollten, gerade in Notfallsituationen, durch die Schaffung von unterstützenden Ressourcen und Systemen gestärkt werden, welche entlang der **zielsetzungs- und erregerspezifischen Prioritäten** optimiert werden. **Ebenso wichtig ist eine Harmonisierung innerhalb von und zwischen verschiedenen Netzwerken und Initiativen.**

(3.) Verbesserung des Datenaustauschs und des Nutzens für optimierte lokale bis globale Entscheidungen und Maßnahmen im Bereich der öffentlichen Gesundheit

Das Ziel konzentriert sich auf die **Interoperabilität von Systemen durch Standardisierung unter Berücksichtigung von Qualität, Transparenz, Austausch und Kollaboration**. Des Weiteren ist es wichtig, dass **wesentliche Metadaten für einen maximalen Nutzen erfasst werden.**

(4.) Maximierung der Verknüpfung für eine rechtzeitige Wertschöpfung in der breiteren Surveillance-Architektur

Die Verknüpfung des Datenflusses innerhalb/zwischen Ländern und Disziplinen sowie auch über verschiedene Erregergruppen hinweg (z. B. bei Multi-Spezies-Ausbrüchen) ist essentiell, um Impact, Ausrichtung und Effizienz zu maximieren. **Genomische Surveillance für die pan- und epidemische Preparedness and Response wird am erfolgreichsten durch die Verknüpfung und Nutzung von existierenden Stärken und Kapazitäten.**

(5.) Erhaltung der Einsatzbereitschaft in Notfallsituationen

Neben der **routinemäßigen Surveillance** ist es ebenso wichtig, die Kapazitäten für Notfallsituationen, wie die Verbreitung neuer Krankheitserreger, **schnell und angemessen skalieren zu können.**
